# Genome-wide identification and characterization of ABA receptor pyrabactin resistance 1-like protein (PYL) family in oat

**DOI:** 10.7717/peerj.16181

**Published:** 2023-10-02

**Authors:** Wenbo Mi, Kaiqiang Liu, Guoling Liang, Zhifeng Jia, Xiang Ma, Zeliang Ju, Wenhui Liu

**Affiliations:** Key Laboratory of Superior Forage Germplasm in the Qinghai-Tibetan Plateau, Qinghai Academy of Animal Husbandry and Veterinary Sciences, Qinghai University, Xining, China

**Keywords:** *Avena sativa* L., PYL family, Genome-wide, Expression patterns

## Abstract

Abscisic acid (ABA) is a phytohormone that plays an important role in plant growth and development. Meanwhile, ABA also plays a key role in the plant response to abiotic stressors such as drought and high salinity. The pyrabactin resistance 1-like (PYR/PYL) protein family of ABA receptors is involved in the initial step of ABA signal transduction. However, no systematic studies of the PYL family in “*Avena sativa*, a genus *Avena* in the grass family Poaceae,” have been conducted to date. Thus, in this study, we performed a genome-wide screening to identify *PYL* genes in oat and characterized their responses to drought stress. A total of 12 *AsPYL* genes distributed on nine chromosomes were identified. The phylogenetic analysis divided these *AsPYLs* into three subfamilies, based on structural and functional similarities. Gene and motif structure analysis of *AsPYLs* revealed that members of each subfamily share similar gene and motif structure. Segmental duplication appears to be the driving force for the expansion of *PYLs*, Furthermore, stress-responsive *AsPYLs* were detected through RNA-seq analysis. The qRT-PCR analysis of 10 *AsPYL* genes under drought, salt, and ABA stress revealed that *AsPYL* genes play an important role in stress response. These data provide a reference for further studies on the oat *PYL* gene family and its function.

## Introduction

Oat (*Avena sativa* L.), one of the most important cereal crops grown worldwide, is prized as one of the richest sources of vitamin B1, plant-based protein, and healthy fat (Gutierrez-Gonzalez, Tu & Garvin, 2013; Mahmoud et al., 2022). In addition, oats play a prominent role in livestock production due to their variety, high yield, and excellent nutritional quality (He & Bjornstad, 2012). Oats are primarily produced in the temperate regions of the northern hemisphere, especially in Canada, Russia, the United States, Germany, Australia, Finland, and China. However, environmental stressors such as drought can severely constrain oat yields (Tajti, Pál & Janda, 2021). Thus, breeding oat varieties with increased drought resistance is of considerable importance and schemes to improve the yield and quality of oat will require the efficient screening of beneficial genes. Water availability is a limiting factor for many crop plants (Seleiman et al., 2021). Drought stress negatively impacts both plant development and yield by dysregulating important physiological and biochemical processes (Fahad et al., 2017). Under drought stress, signal molecules detect and transmit stress signals to activate the expression and regulation of a variety of stress-related genes. This signaling cascade results in a series of adaptive physiological and biochemical changes in order to maintain basal metabolism and dynamic balance with the external environment (Neill et al., 2008). Abscisic acid (ABA), a phytohormone and key signaling molecule, extensively regulates plant growth, development, and stress response, and the ABA content increases rapidly upon exposure to drought, saline-alkali, and other stress conditions (Zhu, 2016). Under abiotic stress, ABA signaling is mediated by the PYL-PP2C-SnRK2 core signaling pathway. The PYL protein family interacts with other proteins, such as phosphatases in the APP2C branch. Several members of the PYL family have been characterized in plants (Dittrich et al., 2019; Park et al., 2009). Structural analyses have defined the ternary crystal features of PYLs, the PP2C proteins, and their substrate ABA (Meskiene, 2004; Santiago et al., 2012).

As a key component of ABA signaling, the specific functions of PYL proteins have been studied in a variety of plants. In *Arabidopsis*, *AtPYL13* and *AtPYL6* have been found to restrain seed germination (Fuchs et al., 2014), and *AtPYR1* and *AtPYL8* have been found to facilitate ABA-mediated root growth, seed germination, and stomatal closure (Gonzalez-Guzman et al., 2012; Zhao et al., 2014). Overexpression of *AtPYL4* increased the drought tolerance of *Arabidopsis* (Pizzio et al., 2013). Moreover, AtPYL6 was found to interact with MYC2, a core jasmonic acid (JA)-responsive transcription factor, effectively linking the ABA and JA signaling pathways to regulate a series of downstream developmental and stress response processes (Aleman et al., 2016). In rice, *OsPYL5*, *OsPYL9*, and *OsPYL10* receptors have been characterized through overexpression, and *PYL*-knockout mutants exhibit enhanced abiotic stress tolerance and yield (Chen et al., 2017; Kim et al., 2014; Verma et al., 2019). In maize, Overexpression of *ZmPYL8*, *ZmPYL9*, and *ZmPYL12* resulted in increased cold tolerance (He et al., 2018). Taken together, these studies indicate that the *PYL* gene family plays an important role in both plant development and abiotic stress response. Identifying the *PYL* genes in oat will be essential to not only better understand ABA signal transduction in this important crop, but also to breed oat varieties with enhanced stress resistance.

Genetically, oat is an allohexaploid species (2n = 6x = 42), with three (A, C, and D) homoeologous subgenomes. These characteristics make oat an ideal model for studies of polyploidization, subfunctionalization, and homoeologous chromosome interactions (Peng et al., 2022). Members of the *PYL* gene family have been successfully identified in many plants, including 14 *PYLs* in *Arabidopsis* (Dittrich et al., 2019), 13 *PYLs* in rice (Yadav et al., 2020), 38 *PYLs* in wheat (Lei et al., 2021), 14 *PYLs* in tomato (Sun et al., 2011), and 29 *PYLs* in tobacco (Bai et al., 2019). However, the *PYL* gene family has not been fully characterized in oat, hampering its application in oat stress improvement. In this article, we identified members of the *PYL* gene family in oat and analyzed the role of these *AsPYLs* in drought resistance. The results of this study will provide a theoretical basis for the development of oat varieties resistant to drought.

## Materials and Methods

### Identification of the PYL genes in *Avena sativa*

The oat HMM file and proteins sequence of the PYL domain (PF10604) were obtained from the grain genes database (https://wheat.pw.usda.gov/) (Wang, Wang & Paterson, 2012) and Pfam database (https://ebi.ac.uk/Tools/pfa/pfamscan/) (Finn et al., 2000), respectively. According to the HMMER file to search, the *PYL* genes in the oat proteins. All candidate genes were further confirmed by the Pfam, CDD (https://www.ncbi.nlm.nih.gov/cdd/) (Lu et al., 2019), and SMART (http://smart.embl-heidelberg.de/) (Jrg et al., 2000). Then, we also studied the Mw and pI of all found genes by Expasy (https://web.expasy.org/compute_pi/) (Panu et al., 2012). Furthermore, the subcellular localization of these genes was also identified by Cell-PLoc (http://www.csbio.sjtu.edu.cn/bioinf/Cell-PLoc-2/) (Horton et al., 2007).

### Phylogenic and conservative motif analysis of PYL family members

Multiple sequence alignments of the AsPYL protein domain were performed using MEGA 7. Then, phylogenetic reconstruction based on the PYL domain of Arabidopsis, maize, wheat, rice and oat were obtained using the neighbor-joining method, and the program parameters were as follows: p-distance, 80% cutoff of partial deletion, and 1,000 bootstrap repeats. The conserved motifs of all AsPYL proteins were studied using MEME (https://meme-suite.org/meme/) (Bailey et al., 2009), and the default parameters were as follows: the number of motifs was 10, with the optimum motif width ranging from 6 to 50. The exon-intron structure of the *AsPYL* genes were acquired from the genome annotation file using TBtools software (Chen et al., 2020). The upstream 2,000 bp sequences of all *AsPYL* genes were screened using PlantCare (https://bioinformatics.psb.ugent.be/webtools/plantcare/html/) to identify the *cis-*elements of the promoter (Lescot, 2002).

### Chromosomal location and synteny analysis of PYL genes

According to the oat genome annotation file, TBtools shows chromosome distribution and mapping of all *AsPYL* genes (Chen et al., 2020). The multicollinearity Scan toolkit (MCScanX) with default parameters was used to analyze the gene repetition events (Wang et al., 2012). To show the synteny of orthologous PYL genes acquired from oat with maize, Arabidopsis, rice, and wheat, a synteny analysis plot was constructed using TBtools (Dual Synteny Plotter).

### Expression analysis of AsPYL under various conditions

Expression data of *AsPYL* under stress and drought conditions was calculated based on previous study (Liu et al., 2022; Wu et al., 2017). Moreover, this studied analyzed transcriptome data under various stress conditions. Gene expression and heat maps were performed using TBtools software (Chen et al., 2020).

### Protein interaction network of AsPYLs

The protein interaction network of AsPYLs were predicted by GeneMAINA (http://genemania.org/) and String (https://www.string-db.org/; medium confidence 0.400), based on maize orthologous proteins. The protein–protein interaction (PPI) network and node network diagrams were constructed using the Cytoscape software 3.2 (Institute for Systems Biology, Seattle, WA, USA) (Shannon, 2003).

### Plant material, growing conditions, drought and salt treatments

Oat seeds were sown in the soil in plastic tanks and grown in greenhouses. After 2 weeks, the seedlings were acclimated to growth chamber conditions for 48 h and further treated in 200 mM NaCl solutions, 10% PEG6000, and 100 μM ABA for 0, 2, 4, 8, 12, and 24 h, respectively. Then, the treated seedlings were then harvested for RNA extraction and stored at −80 °C.

### qRT-PCR analysis

The total RNA was extracted using the plant RNA Kit (Takara, Shiga, Japan), the cDNA was synthesized using a PrimeScript™ RT reagent Kit (Takara, Shiga, Japan). The 10 *AsPYL* genes were selected to validate the expression level under salt and drought stress and primers, as listed in Supplemental File 1. qRT-PCR was carried out on Light Cycle96 with TB Green® Premix Ex Taq™ II (Takara, Shiga, Japan), the PCR conditions was conducted as follows: 30 s at 95 °C, 40 cycles of 95 °C for 5 s and 60 °C for 40 s. The relative expression levels of AsPYL genes were analyzed by the 2^−ΔΔCT^ method (Livak & Schmittgen, 2013).

## Results

### Identification of PYL gene family members in *Avena sativa*

**Figure 1:**
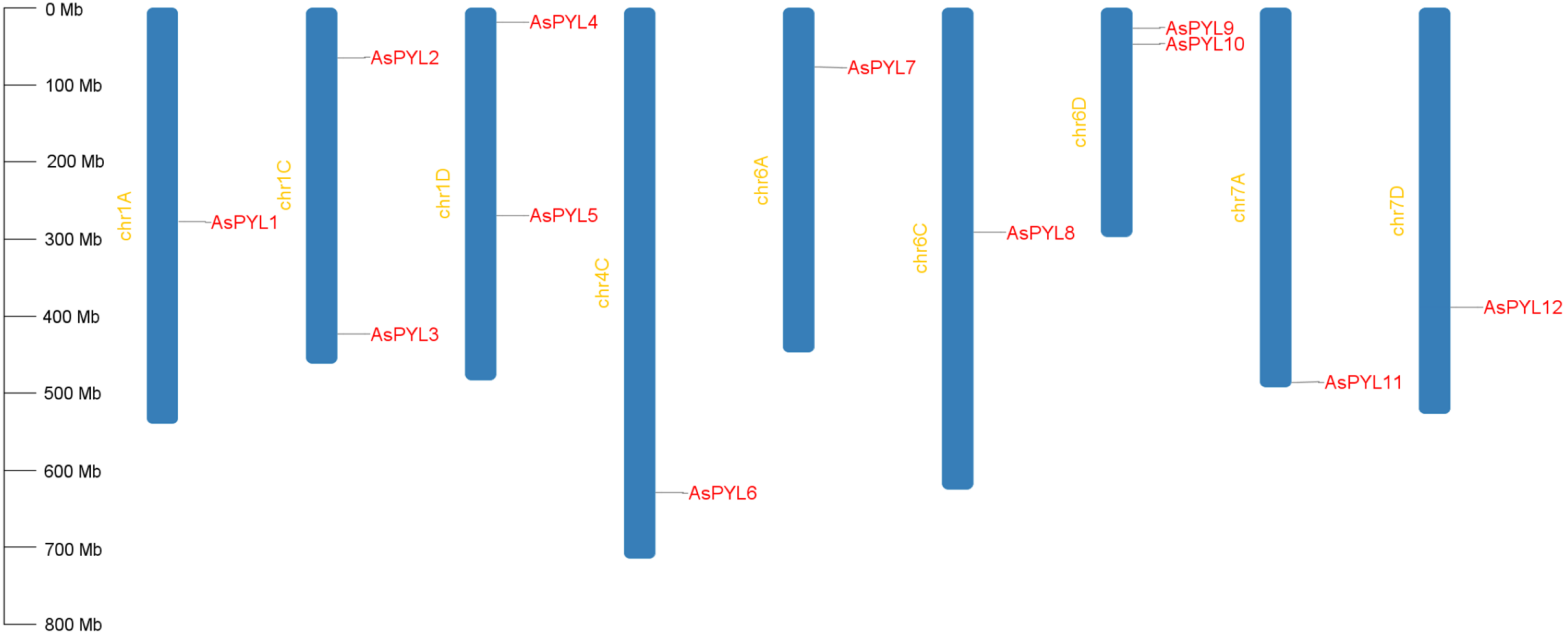
Chromosomal location of *AsPYL* genes.

A total of 12 *PYL* genes were identified in oat, which were distributed in nine out of 21 oat chromosomes (Fig. 1). Chromosomes 1C, 1D, and 6D had the highest abundance of *PYL* genes (two each), followed by 1A, 4C, 6A, 6C, 7A, and 7D (one each). No *PYL* genes were identified on chromosomes 2, 3, or 5. In total, 3, 4, and 5 *AsPYL* genes were identified in the A, C, and D subgenomes, respectively. These results suggest no significant differences in *PYL* gene abundance at the subgenome scale.

The chemical and physical properties of the *PYL* genes and PYL proteins, including isoelectric point (pI), gene length, open reading frame (ORF) length, exon number, amino acid (aa) length, and molecular weight (MW), were estimated using the ExPASY website. The AsPYLs ranged in length from 201 to 254 aa, with an average of 222 aa. The MW of the AsPYLs ranged from 21.65 to 26.92 kDa, with an average of 24.05 kDa. The predicted pIs of the AsPYLs ranged from 5.07 to 9.90, with an average of 6.29. Eleven of the AsPYL proteins were predicted to be localized to the cytoplasm and chloroplasts, with only one AsPYL extracellularly localized (Table 1).

**Table 1:** Basic information of AsPYL family genes and their proteins in *Avena sativa*.

Gene	Gene ID	Gene length (bp)	ORF length (bp)	No. of exon	Predicted protein			Subcellular localization
					Size (aa)	MW (kDa)	pI	
AsPYL1	AVESA.00001b.r3.1Ag0000971.2	1,109	738	1	245	25.89	5.59	Chloroplast
AsPYL2	AVESA.00001b.r3.1Cg0000613.2	2,045	618	3	205	21.97	5.60	cytosol
AsPYL3	AVESA.00001b.r3.1Cg0001670.2	914	723	1	240	25.39	5.31	Chloroplast
AsPYL4	AVESA.00001b.r3.1Dg0000157.1	2,030	618	3	205	21.97	5.61	cytosol
AsPYL5	AVESA.00001b.r3.1Dg0000962.2	1,386	738	1	245	25.78	5.47	Chloroplast
AsPYL6	AVESA.00001b.r3.4Cg0003243.2	1,274	765	1	254	26.41	8.2	Chloroplast
AsPYL7	AVESA.00001b.r3.6Ag0000500.3	3,461	726	2	241	26.92	9.9	cytosol
AsPYL8	AVESA.00001b.r3.6Cg0002306.1	3,545	642	3	213	23.71	6.26	cytosol
AsPYL9	AVESA.00001b.r3.6Dg0000148.1	961	609	1	202	21.65	5.07	cytosol
AsPYL10	AVESA.00001b.r3.6Dg0000219.1	3,665	642	3	213	23.79	5.93	cytosol
AsPYL11	AVESA.00001b.r3.7Ag0002908.1	4,683	606	3	201	22.54	6.45	cytosol
AsPYL12	AVESA.00001b.r3.7Dg0001928.1	4,756	606	3	201	22.55	6.06	Extracellular

**Figure 2:**
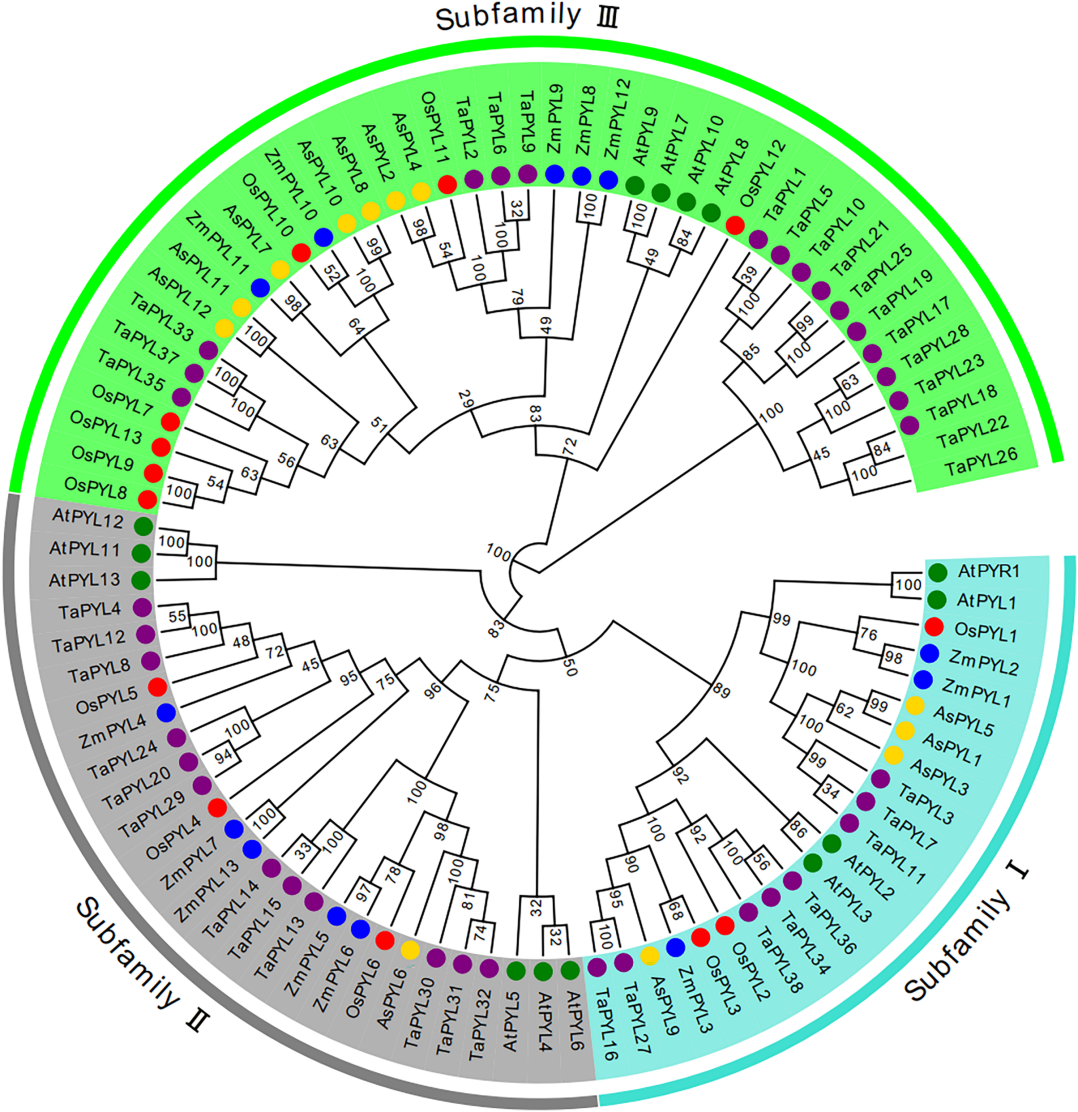
Phylogenetic analysis of *PYL* gene family in *Avena sativa* and its progenitors. Tree was constructed by MEGA7.0 using neighbor-joining method with 1,000 bootstraps. Purple, blue, green, red, and yellow circles represent PYL protein sequences from wheat (Ta), maize (Zm), Arabidopsis (At), rice (Os) and oat (As), respectively. The branch length represents the magnitude of genetic change. Blue, gray and green areas represent subfamilies I, II and III, respectively.

**Figure 3:**
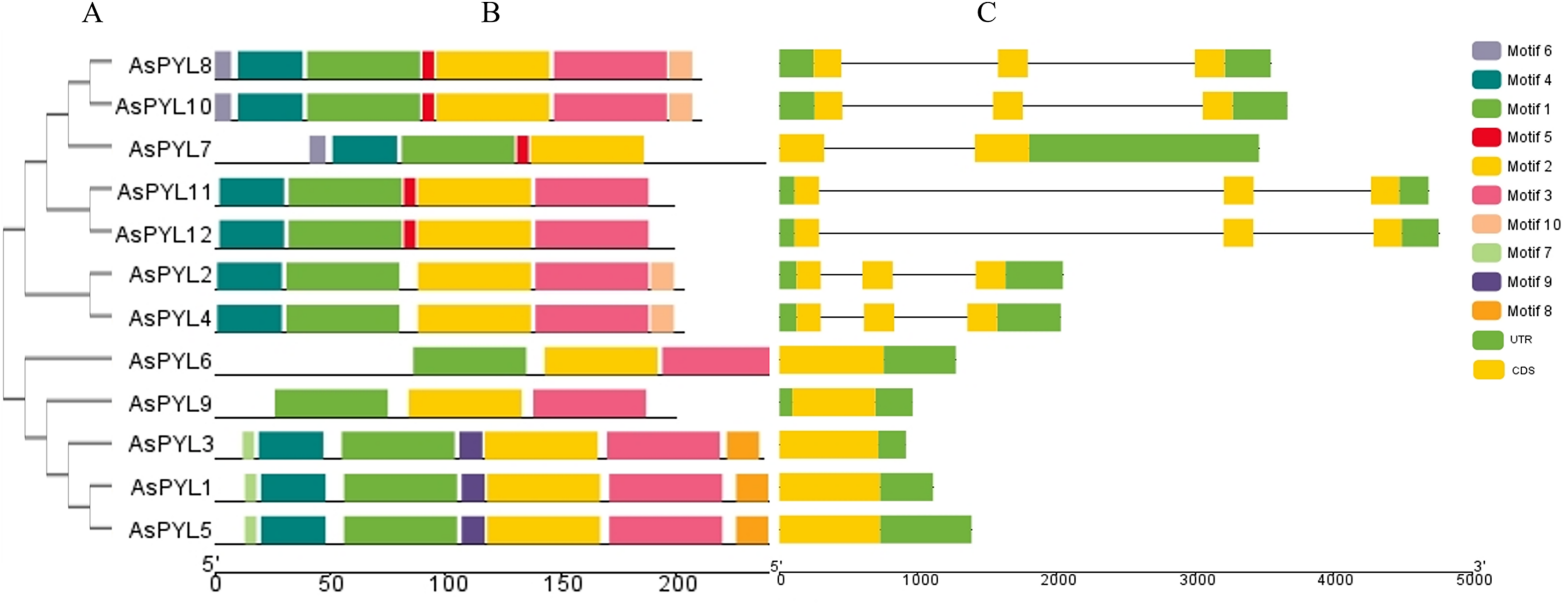
Phylogenetic relationship, conserved motifs and gene structure of AsPYL proteins in *Avena sativa*. (A) The phylogenetic tree was constructed based on 12 AsPYL full-length proteins. (B) The conserved motifs, numbers 1–10, are shown in different colored boxes. (C) The exon-intron structure of *AsPYL* genes. Yellow boxes indicate exons, green boxes indicate untranslated region (UTR) and black lines represent introns.

A phylogenetic tree was constructed based on the 12 AsPYL proteins using MEGA 7 software. According to sequence similarity, the AsPYL proteins can be divided into three subfamilies: I, II, and III (Figs. 2 and 3A). Among the 12 AsPYL proteins, four (AsPYL1, AsPYL3, AsPYL5, and AsPYL9) belong to subfamily I, one (AsPYL6) belongs to subfamily II, and seven (AsPYL2, AsPYL4, AsPYL7, AsPYL8, AsPYL10, AsPYL11, and AsPYL12) belong to subfamily III.

### Comparison to PYL gene family members in other species

To clarify the phylogenetic relationships between oats and other species, a neighbor-joining tree was constructed using the full-length protein sequences of 38 *TaPYLs*, 13 *ZmPYLs*, 14 *AtPYLs*, 13 *OsPYLs*, and our 12 identified *AsPYLs* (Fig. 2). Consistent with previous studies in *Arabidopsis* and wheat, all studied *PYLs* were found to be classified into three subfamilies. In total, subfamily I contained 22 genes (eight *TaPYLs*, three *ZmPYLs*, four *AtPYLs*, three *OsPYLs*, and four *AsPYLs*), subfamily II contained 24 genes (12 *TaPYLs*, five *ZmPYLs*, three *AtPYLs*, three *OsPYLs*, and one *AsPYL*), and subfamily III contained 44 genes (18 *TaPYLs*, five *ZmPYLs*, seven *AtPYLs*, seven *OsPYLs*, and seven *AsPYLs*). In addition, the AsPYLs were found to be most closely related to the TaPYLs, suggesting a close genetic relationship between oat and wheat.

### Gene structure and conserved motifs of AsPYL genes

The conserved motifs of PYL proteins were studied using the MEME program, and 10 motifs were identified across all 12 AsPYL proteins (Fig. 3B). Motifs 1, 2, and 3 were conserved across all PYLs, while motif 4 was conserved among all PYLs except AsPYL6 and AsPYL9. Overall, two AsPYLs (AsPYL 6 and 9) were found to contain three motifs, five AsPYLs (AsPYL2, 4, 7, 11, and 12) were found to contain five motifs, and five AsPYLs (AsPYL1, 3, 5, 8, and 10) were found to contain seven motifs. Although the AsPYLs contained many conserved motifs, each subfamily member was found to also contain unique motifs. These results suggest that while functional similarities exist among members, the presence of unique motifs is indicative of specialized biological functions.

The exon-intron structures of *AsPYL* genes were also analyzed (Fig. 3C), and all 12 *AsPYLs* contained either one (*AsPYL1, 3, 5, 6*, and *9*), two (*AsPYL7*), or three (*AsPYL2, 4, 8, 10, 11*, and *12*) exons. Interestingly, no introns were identified in the subfamily I or II *AsPYL* genes. All members of subfamily III, except *AsPYL7* (one intron), contained two introns. In general, *AsPYL* genes belonging to the same subfamily tended to share similar exon-intron structures, affirming their close evolutionary relationships.

### Synteny analysis of AsPYL genes

**Figure 4:**
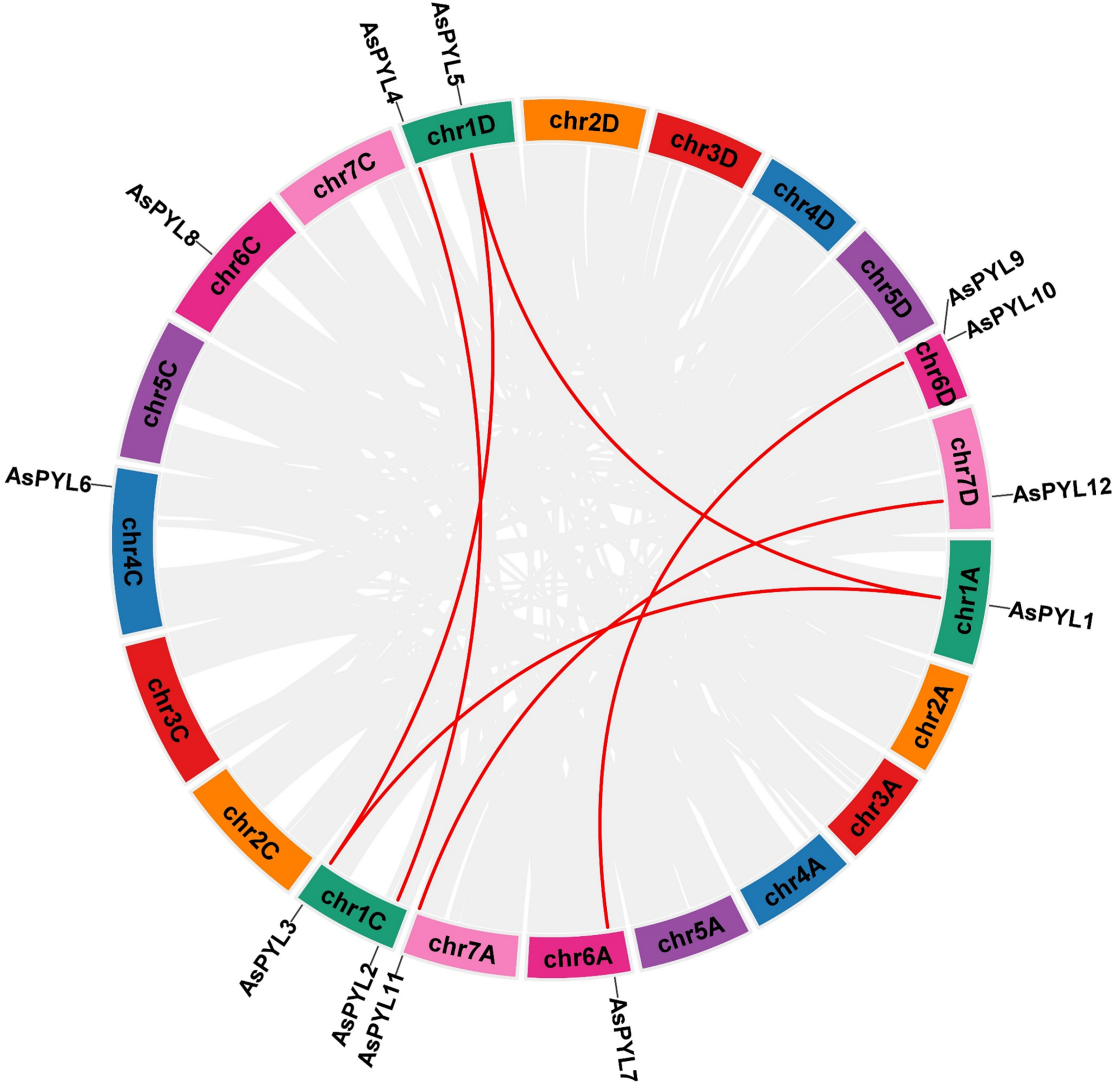
Synteny analysis of *AsPYL* genes family in *Avena sativa*. The gray line represents the syntenic blocks in the oat genome, and the red lines represent segmental duplication gene pairs. Chromosomes 1–7 are shown in different colors.

**Figure 5:**
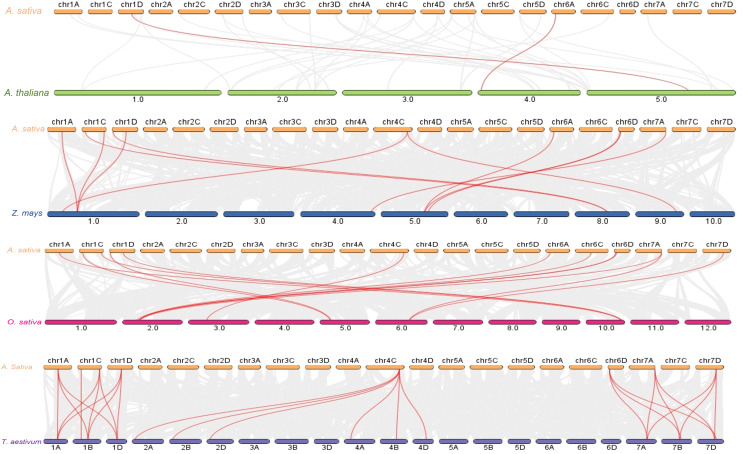
Syntenic analysis of *PYL* gene family among genomes. Gray lines in the background represent collinear blocks within the genome of *Avena sativa* and other plants, while red lines highlight represents the collinearity of *PYL* gene pairs. The species were *A. thaliana*, *Z. mays, O. sativa* and *T. aestivum* respectively.

The increase in evolutionary raw materials resulting from gene replication can facilitate evolution (Cannon et al., 2004; Kong et al., 2007). Here, we analyzed fragment duplications and tandem duplication events in oats. As shown in Fig. 4, we identified six pairs of segmental duplications: *AsPYL4* and *AsPYL2*, *AsPYL5* and *AsPYL3*, *AsPYL5* and *AsPYL1*, *AsPYL1* and *AsPYL3*, *AsPYL7* and *AsPYL10*, and *AsPYL11* and *AsPYL12*. To further study the evolutionary mechanism among *PYL* gene family members, we established a collinearity relationship with the four model plants *Arabidopsis*, rice, maize, and wheat (Fig. 5). The number of syntenic genes between oat and other species was as follows: 25 orthologous pairs between eight *AsPYLs* and 16 *TaPYLs*, 13 paired collinearity relationships between 12 *AsPYLs* and 7 *OsPYLs*, 11 pairs between 10 *AsPYLs* and seven *ZmPYLs*, and two pairs between *Arabidopsis* and oat. In total, we found 11 common *AsPYL* genes among these collinearity relationships. In addition, some *AsPYLs* were found to be associated with the presence of at least two syngenetic pairs (particularly between oat and wheat). For example, two or more homologous pairs contained seven genes (87.50%) in wheat, one (8.33%) in rice, and one (10%) in maize. These genes might play a key evolutionary role.

### Structural analysis of AsPYL gene promoters

**Figure 6:**
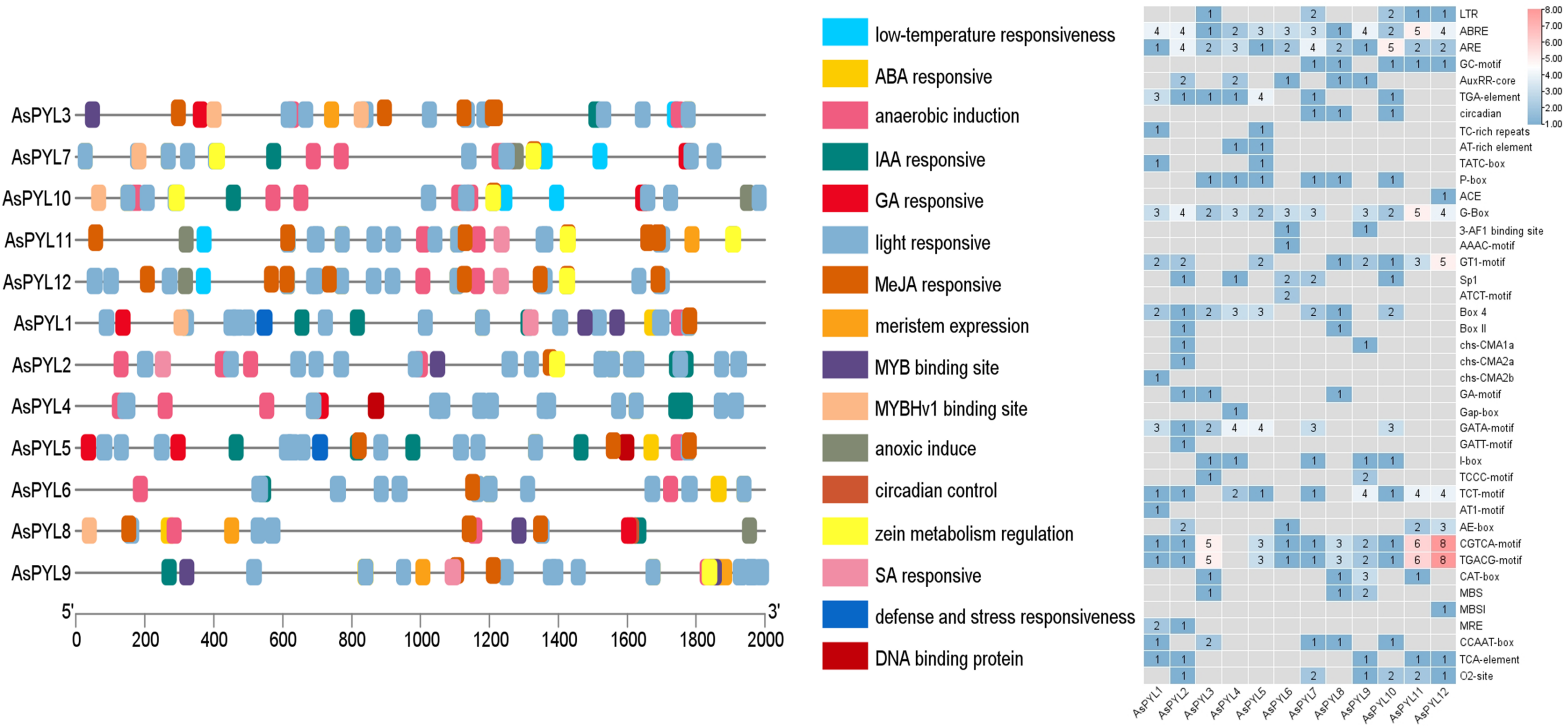
Putative cis-elements existed in the 2 kb upstream region of *Avena sativa* *PYL* genes. (A) The elements which respond are displayed in differently coloured boxes. (B) The number of *cis*-elements present on the Oat *PYL* gene.

In order to better understand the transcriptional regulation and function of the *AsPYL* genes, we predicted the *cis-*regulatory elements in their promoters. A total of 41 functional *cis-*elements were identified. The CGTCA-motif, TGACG-motif, G-box, and other key promoter elements were found in all 12 *AsPYLs*. In addition, a large number of phytohormone-responsive promoter elements were identified among the *AsPYLs*, as well as those involved in growth and development, signal transduction, and abiotic stress (Fig. 6). Among them, ABA-responsive element (ABRE) and anaerobic-responsive element (ARE) were identified in all 12 *AsPYLs*. In addition, methyl-jasmonate (MeJA)-responsive motifs (TGACG and CGTCA) were identified in 11 *AsPYLs*, gibberellin (GA)-responsive motifs (TATC-box and P-box) were identified in eight *AsPYLs*, light-responsive element (LRE) G-box was identified in 11 *AsPYLs*, auxin (Aux)-responsive element (TGA) was identified in seven *AsPYLs*, salicylic acid (SA)-responsive element (TCA) was identified in 5 *AsPYLs*, and ethylene (ET)-responsive element (ERE) was identified in 8 *TaPYLs*. Several stress-related elements, including low temperature-responsive (LTR) MYB binding sites and defense- and stress-responsive TC-rich repeats, were also found in *AsPYLs*. These *cis*-acting elements assist or act on *PYL* genes and form PYL-involved plant regulatory networks, and may play key roles in the oat response to abiotic and biotic stressors.

### Gene Ontology and Kyoto Encyclopedia of Genes and Genomes analyses of AsPYL proteins

The functions of AsPYL proteins were predicted by Gene Ontology (GO) analysis. In the molecular functions category, the proteins were found to be associated with protein phosphatase inhibitor activity (GO0004864), molecular function inhibitor activity (GO0140678), protein dimerization activity (GO0046983), and monocarboxylic acid binding (GO0033293). In the cellular components category, the proteins were found to be associated with the nucleus (GO0005634), cytosol (GO0005829), cytoplasm (GO0005737), and intracellular membrane-bounded organelle (GO0043231). In the biological process category, the proteins were found to be associated with negative regulation of protein serine/threonine phosphatase activity (GO1905183), cellular response to alcohol (GO0097306), regulation of phosphatase activity (GO0010921), and negative regulation of phosphatase activity (GO0010923). Kyoto Encyclopedia of Genes and Genomes (KEGG) analysis indicated that the AsPYL proteins were primarily enriched in plant hormone signal transduction, signal transduction and environmental information processing, and MAPK signaling pathway-plant (Supplemental Files 2 and 3).

### Protein interaction network of AsPYLs

**Figure 7:**
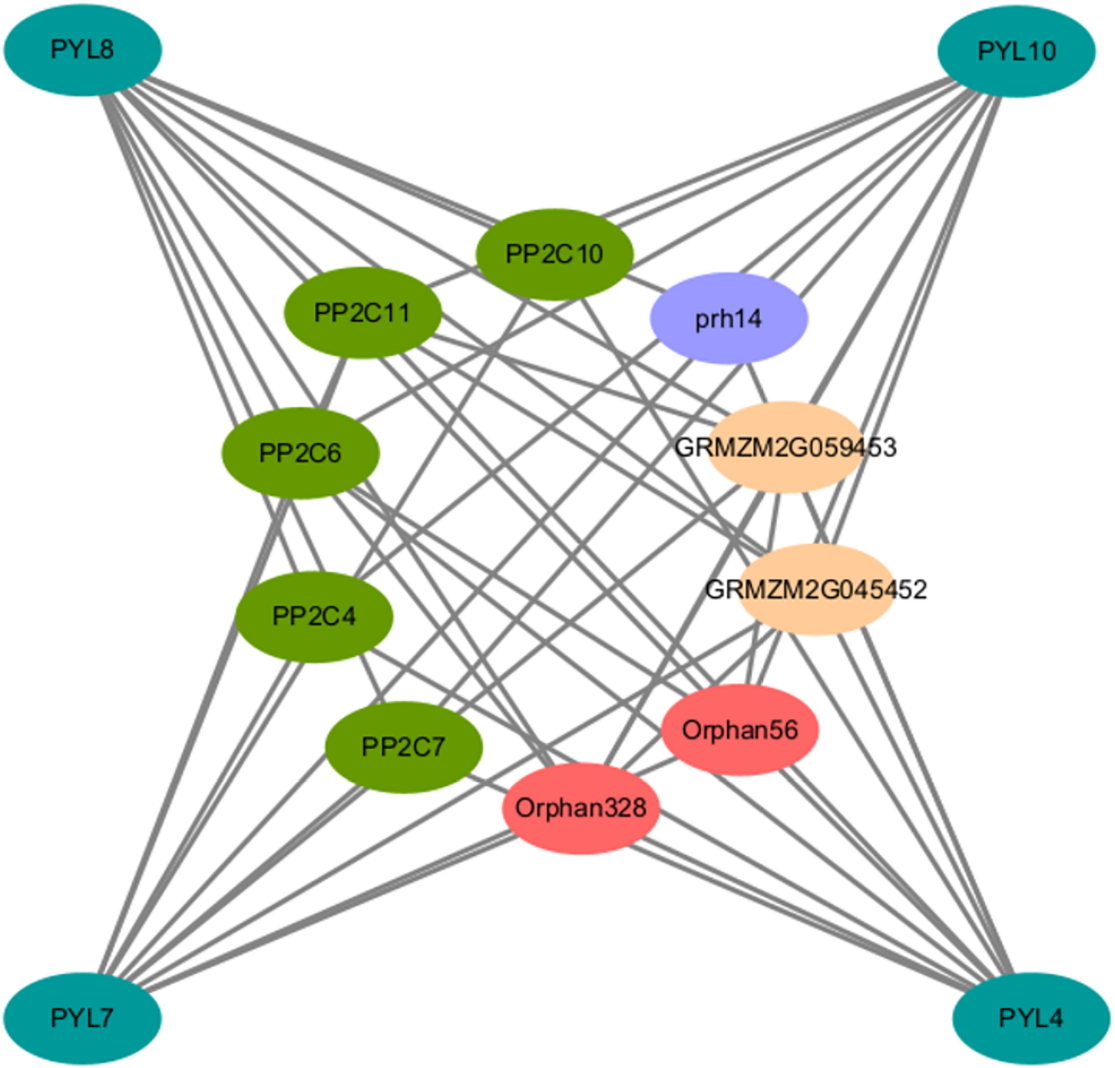
Predicted protein-protein interaction networks of AsPYL proteins with other proteins using STRING tool. The green circles represent oat PYL proteins, and the circles on the inside represent proteins that interact with AsPYLs. Different colors including dark green, red, yellow, and blue phosphatase 2C family proteins, protein-serine/threonine phosphatase protein, probable protein phosphatase 2C, and RNA helicase, respectively. The two circles connected by the gray line represent the interaction between the proteins.

To elucidate the AsPYL regulatory network, a protein-protein interaction (PPI) network was produced. Overall, 4 AsPYLs had orthologous relationships with ZmPYLs and 10 corresponding interacting functional genes were identified. As expected, most of the proteins interacting with AsPYLs were important and functionally-validated components of the ABA signaling complex, such as PP2C (Fig. 7). Based on functional annotation, the proteins interacting with AsPYLs were divided into four categories, including five phosphatase 2C family proteins (PP2C4, PP2C6, PP2C7, PP2C10, and PP2C11), two protein-serine/threonine phosphatase proteins (Orphan56 and Orphan328), two probable protein phosphatase 2Cs (GRMZM2G045452 and GRMZM2G059453), and one RNA helicase (prh14).

### Expression profiling of AsPYL genes by RNA-seq

**Figure 8:**
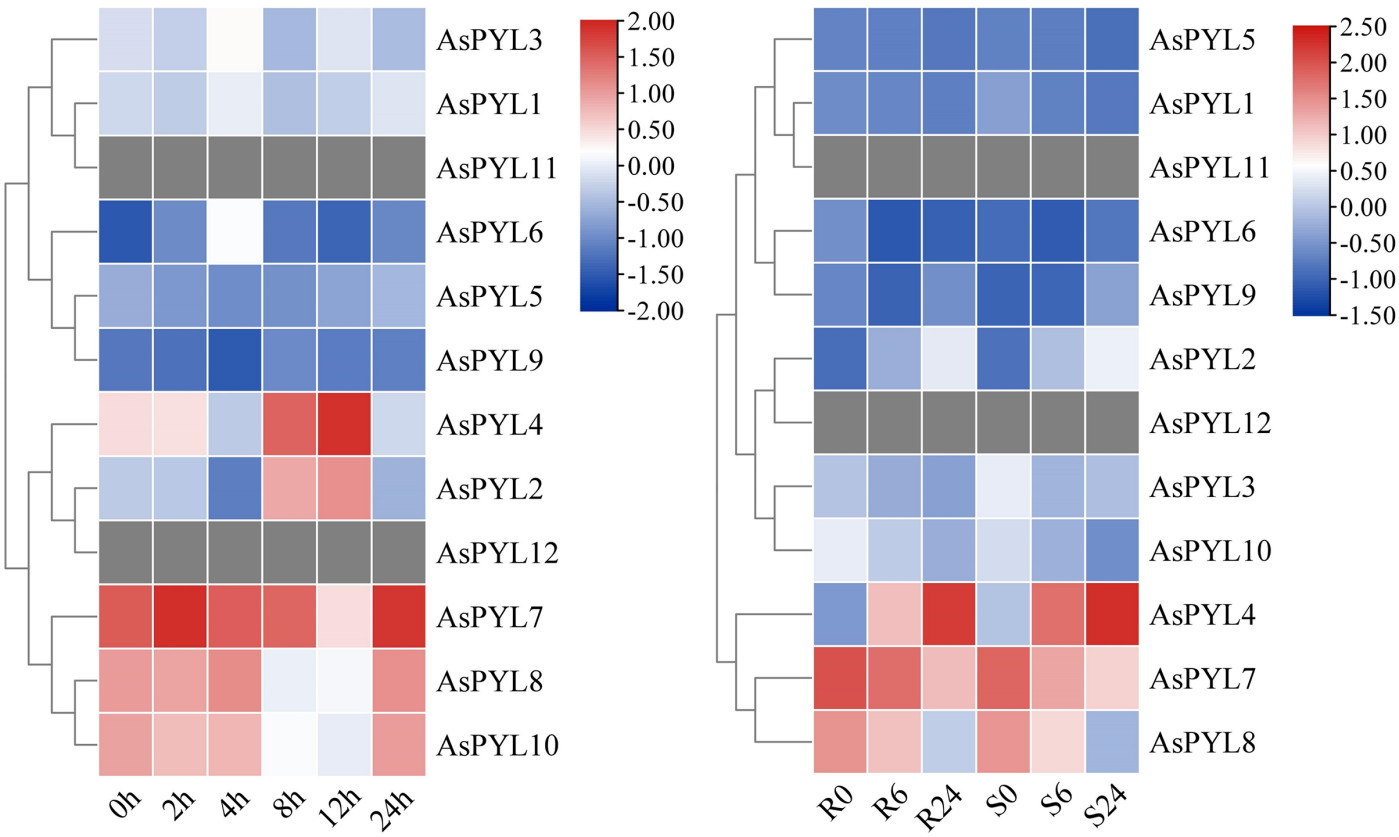
Expression pattern of 12 *AsPYL* genes in different adversity based on RNA-seq data. (A) Gene expression analysis of oat under salt stress at 0, 2, 4, 8, 12, 24 h. (B) The comparison of drought stress between DY2 and MW varieties at 0, 6, and 24 h.

Given the broad regulatory role of the PYL family in plants, we studied the expression patterns of all 12 *AsPYL* genes using publicly-available transcriptome data derived from salt- and drought-stressed oats (Supplemental Files 4 and 5). Notably, not all *AsPYL* genes responded positively to adversity. For example, *AsPYL11* and *AsPYL12* were unexpressed under salt and drought stress treatment. Other *AsPYL* genes were either upregulated or downregulated depending on the treatment, time, and variety. At the same time, the expression levels of different *AsPYL* genes exhibited differences under the same treatment. For example, *AsPYL2*, *AsPYL4*, *AsPYL7*, *AsPYL8*, and *AsPYL10* exhibited higher expression under salt treatment (Fig. 8A), while *AsPYL4, AsPYL7*, and *AsPYL8* exhibited higher expression under drought treatment (Fig. 8B). These results indicate that *AsPYL2*, *AsPYL4*, *AsPYL7, AsPYL8*, and *AsPYL10* may play a significant role in oat drought resistance.

### Expression patterns of AsPYL genes under abiotic stress

**Figure 9:**
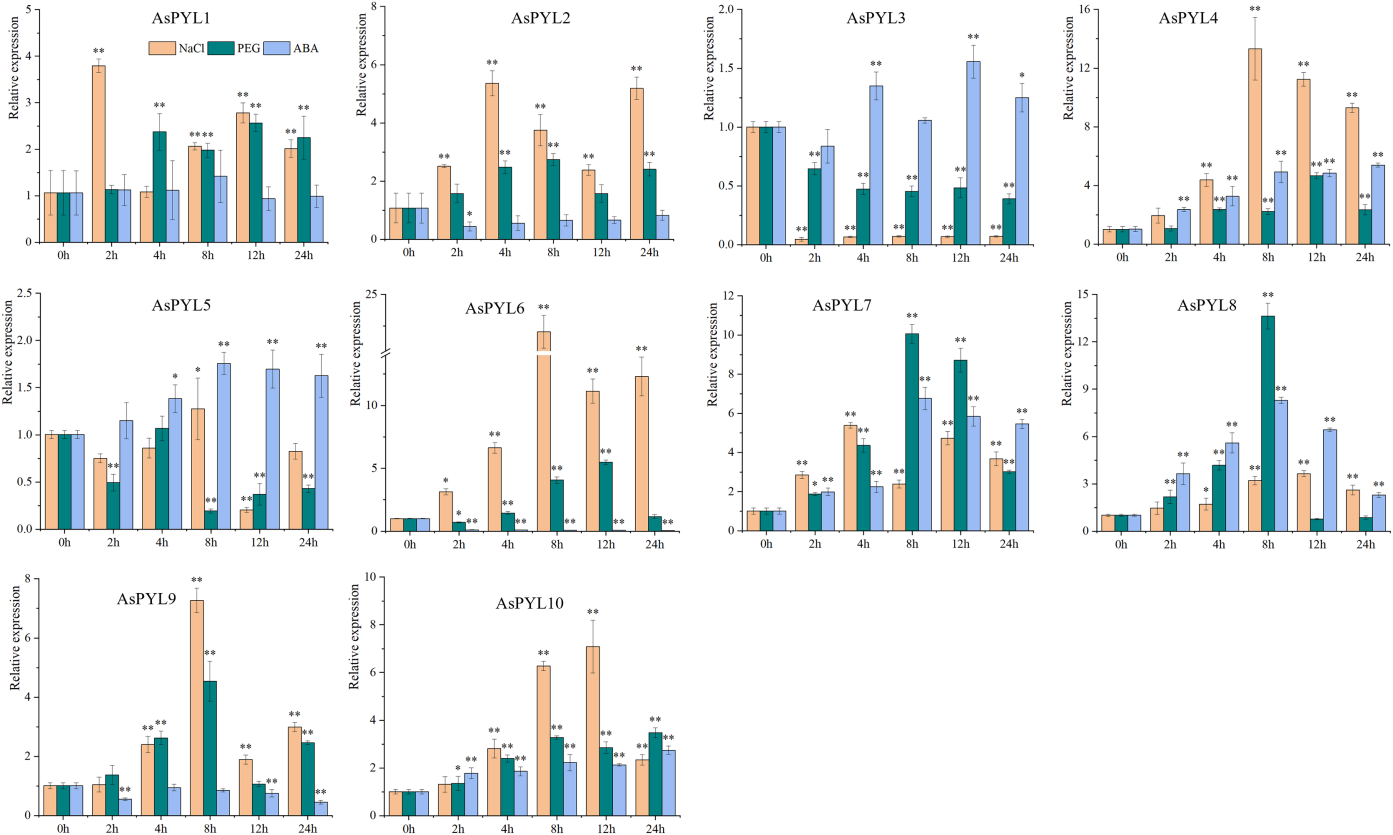
Expression pattern of 10 selected *AsPYL* genes in response to salt, drought, and ABA stress. * indicates significant differences at the 0.05 level, ** indicates extremely significant differences at the 0.01 level.

To examine the expression levels of *AsPYL* genes under drought, salt, and ABA stress, qRT-PCR analysis was performed on 10 *AsPYL* genes (Fig. 9, Supplemental File 6). We found that the majority of the tested *AsPYLs* were induced by drought, salt, and/or ABA treatment. For example, the expression levels of *AsPYL1/2/4/6/7/8/9/10* were significantly up-regulated under salt and drought stress, and the expression levels of *AsPYL3/4/5/7/8/10* were significantly up-regulated under ABA stress. Interestingly, the majority of the up-regulated *AsPYL* genes (*AsPYL2, 4, 7, 8* and *10*) belong to subfamily III, that members of the same subfamily tend to share similar expression patterns. Conversely, the expression levels of *AsPYL3* and *AsPYL5* were inhibited by drought and salt treatment and the expression levels of *AsPYL2, AsPYL6*, and *AsPYL9* were inhibited by ABA treatment.

## Discussion

ABA is an important phytohormone that regulates the plant stress response. Simultaneously, ABA plays an important role in mediating important agronomic traits such as seed germination and maturation and physiological responses to abiotic stress (Finkelstein, Gampala & Rock, 2002; Sun et al., 2011; Xiong, Schumaker & Zhu, 2002; Zhu, 2016). Recently, the discovery of the RCAR/PYL family of ABA receptor proteins served as a breakthrough in our understanding of ABA signaling (Park et al., 2009; Ma et al., 2009). Specifically, PYL receptors play an indispensable role in the initiation of ABA signaling pathways. The function of the *PYL* gene has been characterized in many model species, including *Arabidopsis* (Dittrich et al., 2019), rice (Yadav et al., 2020), and tobacco (Bai et al., 2019). However, there are few studies on the *PYL* gene family in oat.

In this study, we identified 12 *AsPYL* genes and analyzed their basic structure. The genes were named *AsPYL1* to *AsPYL12* based on their chromosomal locations. Based on phylogenetic analysis, the *AsPYL* family can be classified into three subfamilies: I, II and III (Figs. 2A and 3). Subfamily I and II *AsPYLs* are intronless, although all identified subfamily III *AsPYLs* have introns (Fig. 2C). The intron/exon structure and exon number of *AsPYL* genes are similar to those in maize (He et al., 2018), wheat (Lei et al., 2021), rice (Yadav et al., 2020), and cotton (Zhang et al., 2017), indicating that the quantity and structure of exons and introns are reflective of phylogenetic relationships (Fig. 3). Introns play an important role in the post-transcriptional regulation of gene expression through both splicing-independent and splicing-dependent intron-mediated mRNA accumulation (Gallegos & Rose, 2019). All of the subfamily III *AsPYL* members have evolved introns to improve their regulatory activity. In order to study the diversity of the AsPYL proteins, their conserved motifs were identified using the MEME program (Fig. 2B). All 12 AsPYL proteins contained motifs 1, 2, and 3, indicative of the greatly conserved function of this protein family. Phylogenic analysis of *AsPYL* genes revealed that genes within each subfamily tended to contain the same motifs. For example, motifs 7, 8, and 9 were specific to subfamily I members, with the exception of *AsPYL9*. Motifs 5, 6, and 10 were endemic to most subfamily III members. Although *AsPYL* genes of the same subfamily likely have similar biological functions, their specific functions remain to be elucidated. Gene duplication events can be generally divided into five types, including tandem duplication (TD), whole-genome duplication (WGD), proximal duplication (PD), transposed duplication (TRD), and dispersed duplication (DSD), all of which promote gene family expansion in eukaryotes (Doerks et al., 2002; Moore & Purugganan, 2003). WGD events can produce a large number of duplicate genes in a short time (Wang, Wang & Paterson, 2012). WGDs are considered a major feature of eukaryotic genomes, and play an important role in genomic and genetic evolution (Moore & Purugganan, 2003). Several gene families, such as the F-box and SWEET families and heat-shock transcription factors, expanded primarily through WGD and DSD (Li et al., 2016; Qiao et al., 2015; Wang, 2018). However, the expansions of the *AP2/ERF* and *WRKY* gene families mainly resulted from TD events (Guo et al., 2014). In this study, we analyzed gene duplication events in oats, and found that six pairs of gene experienced WGD during genetic evolution. Meanwhile, we evaluated a collinearity relationship between *Arabidopsis*, maize, rice, wheat, and oat (Fig. 5). Collinear gene pairs between oat and maize, rice, and wheat *PYL* genes were most common on oat chromosomes 1, 6, and 7; maize chromosomes 1 and 5; rice chromosomes 2 and 10; and wheat chromosomes 1, 2, 4, and 7. Some of the *PYL* genes exhibited multiple collinearity. Further studies on collinearity of oat and wheat showed that most collinear genes belong to subfamily (Additional File 7). These results further demonstrate that the *AsPYL* genes in subfamilies I and II exhibit higher conservation compared to those in subfamily III.

*Cis*-acting elements, regulatory motifs which act as transcription factor DNA binding sites, play a core role in gene transcriptional regulation (Kao, Cocciolone & Mccarty, 1996; Pl et al., 2020). When plants experience biotic or abiotic stress, transcription factors are activated and bind to *cis*-acting elements to promote the expression of related stress-responsive genes (Xing et al., 2018). Furthermore, *cis*-acting elements are significant regulators of phytohormone responses related to both growth and defense (Zhang et al., 2022). In this study, the *AsPYL* genes were found to be enriched in light-responsive and phytohormone-responsive (ABA, IAA, GA and SA) modules such as ABRE, ARE, TGACG-motif, CGTCA-motif, and G-box. In particular, ABRE and ARE elements are present in all *AsPYL* genes. ABRE elements exist in the promoter sequences of many ABA-inducing genes, and play a vital role in salt tolerance and drought resistance (Pla et al., 1993). We infer that the *AsPYL* genes may take part in the regulation of oat development and defence response. Studies have shown that overexpression of *PYL* genes improves stomatal regulation, seed germination, seedling growth, and drought resistance in *Arabidopsis* (Saavedra et al., 2010; Santiago et al., 2009). In rice, the ABA receptor *OsPYL* has been found to regulate ABA signaling, including improving the sensitivity of seeds to ABA and increasing the drought and salt-alkali stress resistance of rice (Kim et al., 2012; Kim et al., 2014). These results are in agreement with our study, suggesting that *AsPYL* genes take part in the stress defense response through phytohormone signaling pathways.

To evaluate the responses of *AsPYL* genes to various stresses, we obtained publicly available transcriptomic qRT-PCR data on drought- and salt-stressed oats. The majority of *AsPYL* genes were responsive to drought and salt stress, but the expression levels of these genes were not absolutely consistent with the transcriptome data. Therefore, we selected genes involved in drought and salt stress identified in the transcriptome data set for qRT-PCR verification. The results indicate that these genes exhibit high expression levels under drought and salt stress.

## Conclusion

In conclusion, 12 *AsPYL* genes were identified and classified into three subgroups. Their gene structure, phylogenetic relationships, chromosomal locations, conserved motifs, promoter regions, and transcriptomic expression were systematically analyzed. Analysis of *AsPYL* expression patterns and functions indicated that the majority of *AsPYL* genes actively participated in growth, development, and stress response. These data provide a reference for further studies on the oat *PYL* gene family and its function.

### Availability of data

The genome sequence of oat is available in https://wheat.pw.usda.gov/GG3/graingenes-downloads/pepsico-oat-ot3098-v2-files-2021. The 12 AsPYL protein sequences and ids are listed in Supplemental Files 8 and 9 and the sequences of PYLs from Arabidopsis (https://www.arabidopsis.org), maize, rice and wheat genomes (https://plants.ensembl.org/index.html) are available in the respective database. The protein sequences of these species are given in Additional File 6.

## Supplemental Information

10.7717/peerj.16181/supp-1 Supplemental Information 1 Supplementary material.

## Additional Information and Declarations

### Data Availability

The following information was supplied regarding data availability: The raw data is available in the Supplemental Files.
