# Supplementary material for: Genome-wide identification and characterization of ABA receptor pyrabactin resistance 1-like protein (PYL) family in oat
Source: PeerJ. 2023 Oct 2;11:e16181. doi: 10.7717/peerj.16181 (PMC10552766; doi:10.7717/peerj.16181)
Supplement: Supplemental Information 1 [file peerj-11-16181-s001.zip › supplementary material/Supplementary figure or table legends.docx]

**Additional file 1.** qRT-PCR primers of AsPYL genes

**Additional file 2.** *AsPYL* gene family GO annotation information.

**Additional file 3.** *AsPYL* gene family KEGG annotation pathway.

**Additional file 4.** RNA-seq data.

**Additional file 5.** Synteny data

**Additional file 6.** 12 *AsPYL* genes and corresponding ID

**Additional file 7.** Sequence of PYL family gene proteins in Arabidopsis, maize, rice, wheat and Oat

**Additional file 8.** figure 9 raw data

**Additional file 9.** qRT-PCR raw data
