# Supplementary figures and images for: Genome-wide identification and characterization of ABA receptor pyrabactin resistance 1-like protein (PYL) family in oat
Source: PeerJ. 2023 Oct 2;11:e16181. doi: 10.7717/peerj.16181 (PMC10552766; doi:10.7717/peerj.16181)

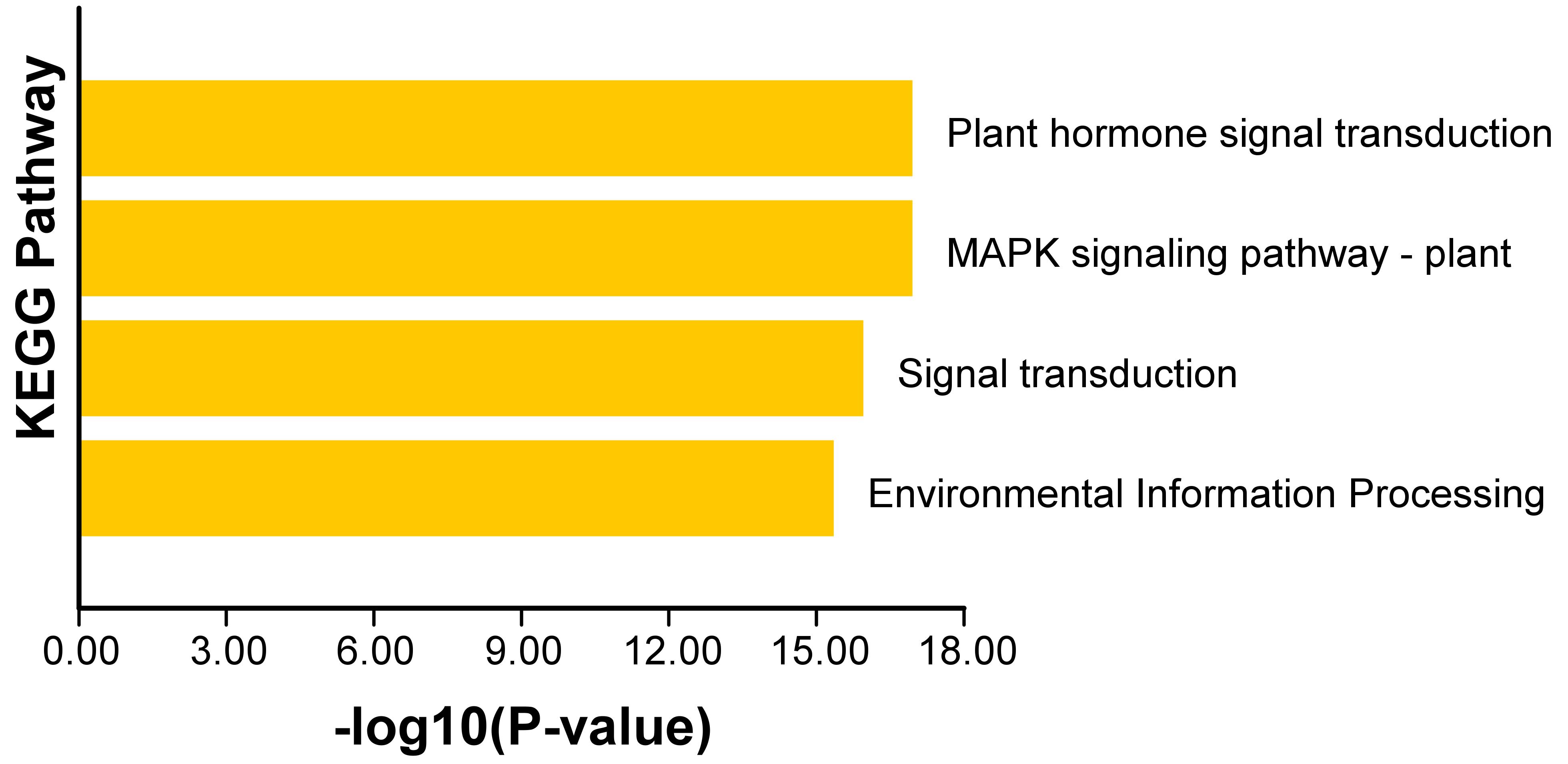

Supplement: Supplemental Information 1 [file peerj-11-16181-s001.zip › supplementary material/Additional file 3.jpg]
